# Adams-Oliver Syndrome: A Clinical Diagnosis in the Genomic Era

**DOI:** 10.7759/cureus.99417

**Published:** 2025-12-16

**Authors:** Srinivas GL, Jignesh Sharma, Sabavath Arun, Ajay Vaidya, Amber Kumar

**Affiliations:** 1 Pediatrics, All India Institute of Medical Sciences, Bhopal, Bhopal, IND; 2 Pediatrics, All India Institute of Medical Sciences, Bhopal,, Bhopal, IND

**Keywords:** adams–oliver syndrome, aplasia cutis congenita, clinical diagnosis, limb defects, vascular disruption, whole-exome sequencing

## Abstract

Adams-Oliver syndrome (AOS) is a rare congenital condition marked by defects of the scalp and malformations of the distal extremities. Although several causative genes have been identified, including ARHGAP31, DLL4, NOTCH1, and DOCK6, a subset of clinically suspected cases remains genetically unresolved. We report the case of a four-year-old female patient, the firstborn of a third-degree consanguineous marriage, who presented with a febrile seizure. Physical examination revealed aplasia cutis congenita (ACC) over the parietal scalp, low-set ears, and bilateral lower limb hypoplasia with absent nails, fulfilling two major diagnostic criteria for AOS. Echocardiography revealed a small ostium secundum atrial septal defect, meeting one minor criterion. Whole-exome sequencing (WES) did not identify any pathogenic variant. The child was referred for surgical evaluation of scalp and limb anomalies and is under multidisciplinary follow-up. Even with breakthroughs in next-generation sequencing, some patients may not have detectable mutations due to genomic heterogeneity, deep intronic or structural alterations, or unidentified new genes. The vascular disruption theory is supported as a likely pathogenic mechanism by the clinical triad of ACC, limb reduction abnormalities, and cardiac anomalies. This example illustrates the continued importance of clinical judgment in diagnosing AOS, even when genomic testing yields conflicting results. Careful phenotypic assessment, guided by established diagnostic criteria, remains essential for appropriate multidisciplinary management and genetic counseling.

## Introduction

Adams-Oliver syndrome (AOS) is a striking constellation of congenital anomalies named after the American pediatricians Frederick H. Adams and R. Oliver, who first described it in 1945. It is a rare multisystem disorder primarily characterized by scalp and skull defects, known as aplasia cutis congenita (ACC), alongside terminal transverse limb defects that can range from brachydactyly to complete aplasia of digits or limbs [1]. The eponym reflects the syndrome's initial recognition in a sibling pair with these hallmark cutaneous and skeletal features, establishing it as a distinct entity amid heterogeneous congenital malformation syndromes. The estimated incidence is one in 225,000 live births, though underreporting is likely due to its variable expressivity and overlap with other disorders [2]. Clinically, AOS warrants suspicion in neonates presenting with bitemporal or vertex scalp erosions/ulcers at birth, coupled with symmetric limb reductions, particularly when accompanied by cardiovascular malformations or subtle neurological signs (e.g., microcephaly or seizures), prompting early multidisciplinary evaluation to guide prognosis and management.

While AOS has been linked to autosomal dominant and recessive mutations in multiple genes, including ARHGAP31, DLL4, NOTCH1, RBPJ, DOCK6, and EOGT, which converge on critical developmental pathways such as Notch signaling (essential for vascular and limb patterning) and Rho GTPase regulation (key for cytoskeletal dynamics and tissue morphogenesis), sporadic cases remain well documented [3,4]. Advances in genomic sequencing have expanded our understanding of AOS, but the persistent genetic heterogeneity and negative molecular findings in a significant portion of patients underscore the critical, enduring role of clinical diagnosis, as illustrated by the following case.

## Case presentation

A four-year-old female child, the firstborn of a third-degree consanguineous marriage, presented in June 2025 with a generalized tonic-clonic seizure lasting one minute, associated with a high-grade fever. A similar febrile seizure had occurred at 2.5 years of age. Developmental milestones were age-appropriate, and family history was unremarkable.

**Figure 1:**
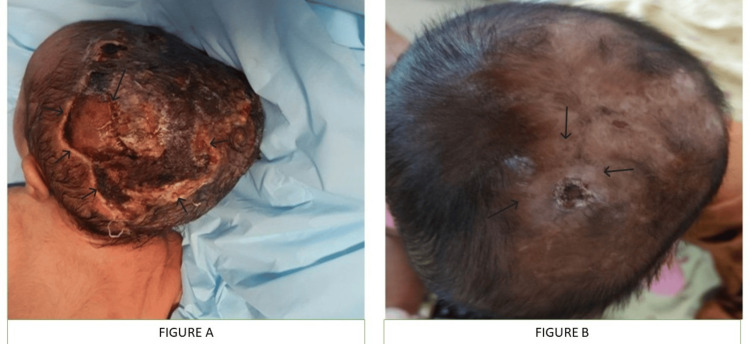
Aplasia cutis congenita over the parietal scalp in the neonatal period (A) and healed scalp scar at four years of age (B).

**Figure 2:**
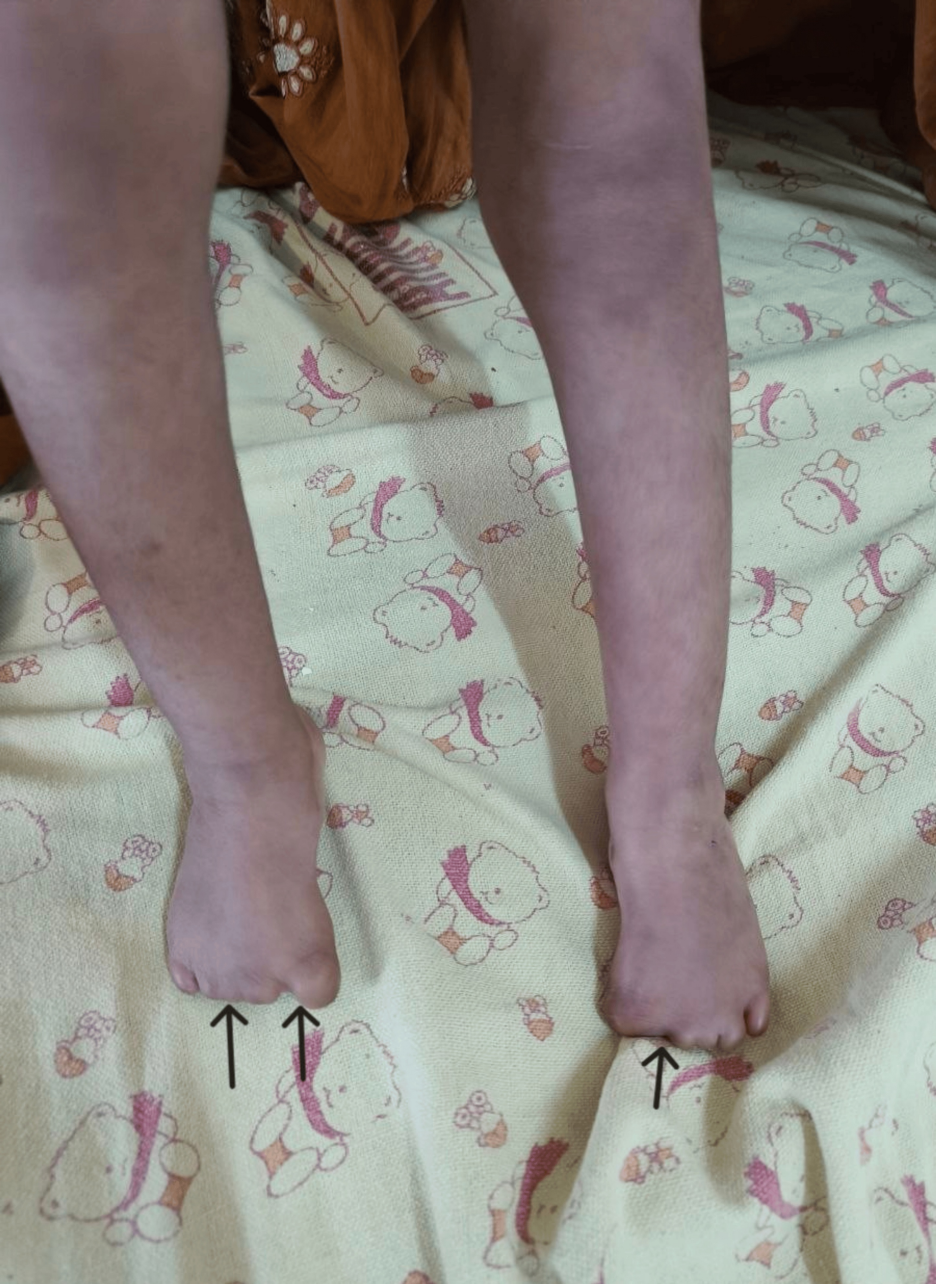
Bilateral terminal limb defects with hypoplastic digits and absent nails involving both lower limbs.

On examination, the child was hemodynamically stable. Dysmorphic features included a 3 × 4 cm area of ACC over the parietal scalp, presenting as alopecia with underlying scarred tissue and no evidence of skull defect on initial cranial ultrasound (Figure 1); low-set ears; and bilateral lower limb anomalies: the second to fifth toes on both feet were severely hypoplastic, appearing as nubs of approximately 5-7 mm in length, with the great toes shorter than expected (approximately 70% of age-matched norms) but better formed; all toenails were absent bilaterally (Figure 2). Based on the combination of ACC and terminal limb anomalies, the child satisfied two major diagnostic criteria for AOS [4]. Cardiac evaluation revealed a small ostium secundum atrial septal defect (4 mm) with a left-to-right shunt and no evidence of right heart volume overload on echocardiography, thus not requiring immediate intervention and fulfilling a minor diagnostic criterion (Table 1). Laboratory investigations for seizure etiology were normal, and neuroimaging (MRI of the brain) and EEG were unremarkable (Table 2).

**Table 1:** Major and minor diagnostic criteria supporting the clinical diagnosis of Adams-Oliver syndrome Reference: [4]

Category	Criteria	Details / Examples
Major Criteria	Terminal transverse limb defects	Hypoplastic digits, absent nails, brachydactyly, ectrodactyly, oligodactyly, or more severe distal limb reduction anomalies.
	Aplasia cutis congenita (ACC)	Most often scalp lesions are noted, may present as solitary or multiple hairless, scarred, or ulcerated areas, sometimes with an underlying bone defect.
	Positive family history	Documented diagnosis of Adams-Oliver syndrome (AOS) in first-degree relatives; supports inherited etiology.
Minor Criteria	Cutis marmorata telangiectatica congenita (CMTC)	Persistent, fixed, mottled vascular skin pattern often involving the limbs.
	Congenital cardiac anomalies	Septal defects and structural malformations such as atrial septal defect (ASD), ventricular septal defect (VSD), or more complex cardiac lesions.
	Vascular anomalies	Pulmonary hypertension, intracranial vascular malformations, renal or hepatic vascular defects.
Diagnostic Threshold	Presence of two major criteria, or one major plus one minor criterion	Confirms a clinical diagnosis of AOS.

**Table 2:** Summary of the laboratory investigations

Parameters	Value	Normal range
Hemoglobin	12	12-16 g%
Total leucocyte count	12400	4000-11000cells/mm3
Platelets	254000	150-450 thousand/microL
C-reactive protein	5.4	<5 mg/dl
Urea	19	20-40 mg/dl
Creatinine	0.4	0.6-1.2 mg/dl
Sodium	138	135-145 mmol/L
Potassium	3.9	3-5 mmol/L
Albumin	3.8	3.5-5.5 g/dl
Blood culture	Sterile	-
Urine culture	Sterile	-
Calcium	8.5	8.8-11 mg/dl
Phosphate	4.2	2.5-4.5 mg/dl
Magnesium	2.4	1.9-2.5 mg/dl

The child was managed conservatively for the acute febrile seizure with antipyretics and hydration, achieving complete resolution of fever and seizure within 48 hours without recurrence during the hospital stay. Given the syndromic presentation and third-degree consanguinity suggesting possible autosomal recessive inheritance, whole-exome sequencing (WES) was performed. WES specifically interrogated known AOS-associated genes (ARHGAP31, DLL4, NOTCH1, RBPJ, DOCK6, EOGT) and identified no pathogenic or likely pathogenic variants, effectively ruling out the most common genetic causes for this presentation. At the three-month follow-up visit in September 2025, the child remained neurologically stable with no further seizures, appropriate weight gain, and stable limb function aided by custom orthotic inserts for gait support. The scalp scar showed complete epithelialization without infection or ulceration. Genetic counseling was provided, and the child was referred to plastic surgery for evaluation of scalp and limb defects, with orthopedic consultation for potential reconstructive options. Multidisciplinary follow-up continues with pediatrics, cardiology (for atrial septal defect (ASD) monitoring), neurology, and genetics every six months to assess for vascular complications and developmental progress.

## Discussion

AOS is characterized by a wide phenotypic spectrum ranging from isolated scalp and limb anomalies to multisystem involvement. The major diagnostic criteria include terminal transverse limb defects, ACC, and a positive family history. In contrast, the minor criteria comprise cutis marmorata telangiectatica congenita, congenital heart anomalies, and vascular malformations (Table 1). Diagnosis is supported when two major criteria or one major plus one minor criterion are fulfilled. Our case met the threshold with two major (ACC and limb anomalies) and one minor criterion (ASD) [2,5].

Our patient's phenotype, featuring a 3 × 4 cm parietal ACC without skull defect, severe bilateral lower limb hypoplasia (second and fifth toe nubs, absent nails), and a hemodynamically insignificant ASD, aligns closely with variants commonly associated with NOTCH1 mutations (emphasizing vascular/limb disruptions) or DOCK6 (recessive, multisystem involvement). Yet, no such variants were detected, underscoring AOS's marked genetic heterogeneity and potential for novel loci. Despite substantial advances in next-generation sequencing, a proportion of clinically diagnosed AOS cases remain genetically unresolved, as in our patient. Given the third-degree consanguinity, an autosomal recessive form of AOS is plausible, particularly involving genes such as DOCK6 and EOGT; however, negative WES findings at these and other loci may reflect limitations in detecting deep intronic, regulatory, or structural variants, or suggest involvement of yet-uncharacterized genes [6]. This emphasizes the continued importance of clinical diagnosis, particularly in resource-limited settings. The most widely accepted pathogenic mechanism remains the vascular disruption hypothesis, in which impaired perfusion during embryogenesis results in scalp and limb abnormalities [7]. Additional systemic features, such as cardiac anomalies, CNS malformations, and developmental delay, are variably present [8].

Management of AOS requires a tailored, multidisciplinary approach to address its multisystem features and prevent complications. For ACC diagnosed at birth, initial conservative treatment is preferred for smaller lesions (<5 cm) without exposed bone, involving moist wound care (e.g., hydrogel or foam dressings), infection prophylaxis (e.g., topical antimicrobials like Betadine), and serial monitoring to promote epithelialization and avoid hemorrhage/meningitis risks; surgical options, such as skin grafting or tissue expansion, are reserved for larger defects or those with underlying skull involvement, as in our case where referral for reconstruction was advised post healing [9]. In this patient with bilateral lower limb hypoplasia and incomplete toes, orthopedic management includes custom orthotics/prosthetics for gait support, physical therapy to enhance mobility, and potential reconstructive surgery (e.g., toe lengthening or syndactyly release if webbing develops); early intervention mitigates functional deficits and improves quality of life. Broader care encompasses cardiology follow-up for ASD monitoring, neurology for seizure prophylaxis, and genetic counseling for recurrence risks (up to 50% in dominant forms, variable in recessive forms). Regular surveillance for vascular complications (e.g., pulmonary hypertension) is essential. This case highlights the enduring role of clinical acumen in diagnosing rare syndromes in the genomic era, particularly when advanced testing fails to provide molecular confirmation [10].

## Conclusions

We report the case of a child with classical features of AOS fulfilling both major and minor diagnostic criteria. Despite a comprehensive evaluation including WES, no genetic variant was detected. This example highlights the limitations of genomic testing and emphasizes the crucial role of clinical assessment in diagnosing, directing interdisciplinary care, and providing family counselling. Key learning includes the following: AOS should be suspected when ACC occurs with terminal limb defects, with or without systemic involvement; the diagnosis can be made clinically by fulfilling established major and minor criteria. Genetic testing, including WES, may not always identify a pathogenic variant, highlighting the continued importance of detailed phenotyping in the genomic era.
